# Conventional and Shared Genetic Association Analysis Between Diabetes Mellitus and Sensorineural Hearing Loss

**DOI:** 10.1155/humu/5099141

**Published:** 2026-09-15

**Authors:** Xiaoyu Wang, Ziyan Zhang, Ke Qiu, Yaxin Luo, Xiaowei Yi, Wendu Pang, Dan Jiang, Jianjun Ren, Yu Zhao

**Affiliations:** ^1^ Department of Oto-Rhino-Laryngology, West China Hospital, Sichuan University, Chengdu, China, scu.edu.cn; ^2^ MRC Integrative Epidemiology Unit, Bristol Medical School, University of Bristol, Bristol, UK, bristol.ac.uk; ^3^ Department of Pathology, West China Hospital, Sichuan University, Chengdu, China, scu.edu.cn

**Keywords:** cross-sectional analysis, diabetes, genetic correlation, sensorineural hearing loss

## Abstract

**Purpose:**

This study aims to investigate the epidemiological and genetic associations between diabetes mellitus (DM) and sensorineural hearing loss (SNHL) across different subtypes.

**Methods:**

We analyzed 502,490 participants from the UK Biobank using multivariate logistic regression to examine the association between DM and SNHL, considering gender, age, and HbA1c levels. Genetic correlations and causality were examined by linkage disequilibrium score regression and bidirectional Mendelian randomization. Cross‐trait meta‐analyses identified shared loci between DM and SNHL, followed by gene annotation, functional analysis, and drug candidate exploration for the shared traits.

**Results:**

Observational analysis revealed significant associations between DM and SNHL, consistent in subgroups based on age, sex, and certain HbA1c levels. A positive genetic correlation was found between type 2 diabetes mellitus (T2D) and SNHL (Rg = 0.0982, *p* = 0.0095) between T2D and SNHL, and four loci were identified, with *ARHGEF28* and *TCF7L2* prioritized as credible pleiotropic genes. Enrichment was indicated in glucose metabolism and organogenesis, with shared heritability in metabolic tissues and outer hair cells. Metformin was identified as potential drug candidates for the T2D‐SNHL comorbidity.

**Conclusion:**

These findings progress our understanding of the epidemiological association, shared genetic basis, and potential therapeutic targets between T2D and SNHL, which might contribute to the management of their comorbidity.

**Highlights**

There is a widespread association between diabetes and SNHL, and type 2 diabetes (T2D) shares a common genetic architecture with SNHL. ARHGEF28 and TCF7L2 are associated with the comorbidity of T2D and SNHL, and metformin may serve as a potential therapeutic agent.

## 1. Introduction

Diabetes mellitus (DM) is a globally highly prevalent metabolic disorder marked by hyperglycemia due to defects in insulin secretion, insulin action, or both [1, 2]. The worldwide prevalence of DM is estimated at 463 million, with its prevalence increasing with age [3, 4]. Type 2 diabetes mellitus (T2D) is the most common form of DM, accounting for 90%–95% of cases [5]. Typically, T2D arises from a genetic predisposition to *β*‐cell susceptibility and ultimately results in *β*‐cell dysfunction due to chronic insulin resistance. Whereas type 1 diabetes mellitus (T1D), which constitutes the majority of the remainder, originates from autoimmune pancreatic destruction of *β*‐cell and usually progresses to absolute insulin deficiency [3, 6].

Sensorineural hearing loss (SNHL), primarily caused by dysfunction of the nerve fibers or auditory sensory cells of the inner ear [1], is emerging as a major health concern among patients with DM. According to the data from the National Health and Nutrition Examination Survey (NHANES), among individuals with DM aged 50‐69 years, more than 70% have SNHL. Hypothesized underlying biological mechanisms include inner ear alterations caused by DM, such as microangiopathy [7], decretion of the neuroganglion cell density in the spiral ganglion [8], downregulation of Na+/K+‐ATPase pump [9], mitochondrial dysfunction in spiral ganglion neurons [10], expansion of the endolymphatic fluid compartment and hydrops [11, 12], atrophic changes of organ of Corti cells [10, 12–15], and so on. Although prior researches have reported on the association between DM and SNHL [12, 16–18], Mendelian randomization (MR) analysis for causality inference between DM and SNHL is still lacking, and there also has been a paucity of subsequent investigations examining the relationship in specific diabetes grades (based on glycated hemoglobin levels) or diverse forms of hearing impairment, including unilateral/bilateral and moderate/severe hearing loss.

To date, some studies have indicated the genetic risk factors in DM [19–22] and SNHL [23–25], and suggested the potential genetic underpinnings of comorbid DM and SNHL [3, 26–28]. However, researches on the shared genetic structure of DM and SNHL are still lacking.

In our study, by leveraging observational and genetic data from large population cohorts, we aim to quantify more specific phenotypic associations and constructed a shared genetic architecture for DM and SNHL, encompassing global and local genetic correlations, potential causal relationships, pleiotropic loci and genes, biological mechanisms as well as potential therapeutic medications.

## 2. Materials and Methods

This study is divided into two parts. The first part focuses on the cross‐sectional clinical phenotype association between DM and SNHL based on UK Biobank data. The second part involves a genetic correlation between DM and SNHL based on data from genome‐wide association study (GWAS) analyses.

### 2.1. Phenotypic Association Analyses Between DM and SNHL

#### 2.1.1. Information of Study Participant From UK Biobank

In this cross‐sectional study, data of participants were available from an open, public access database (https://www.ukbiobank.ac.uk/). UK Biobank is a large‐scale cohort study involving over 500,000 UK participants aged 40–69, who were enrolled between 2006 and 2010 and from whom deep genotypic and phenotypic data were collected [29]. Participants were excluded when meeting the following criteria: (1) without speech‐reception‐threshold (SRT) estimate test data for both ears; (2) with any kind of cancer except non‐melanoma skin cancer; (3) with deaf mutism; (4) with organic diseases of the ear; and (5) withdraw from UK Biobank during follow‐up. The detailed exclusion criteria, including field IDs and International Classification of Diseases (ICD) codes, are presented in Table S1. Eventually, 140,732 participants were included for our analyses.

#### 2.1.2. Definition of DM and SNHL

DM was defined using self‐report, physician diagnosis, insulin use, and ICD records, and was further subclassified into T1D and T2D according to diagnosis‐related clinical information. DM cases were additionally stratified by HbA1c levels. SNHL in the observational analysis was primarily defined using SRT estimates after excluding conductive and mixed hearing loss. According to ear‐specific SRT results, SNHL was classified as total, unilateral, or bilateral, and bilateral SNHL was further stratified by severity. Detailed definitions are provided in the Supplementary Methods.

#### 2.1.3. Information of Covariate

The covariates included age, sex, education background, current employment status, body mass index (BMI), ethnic background, smoking status, alcohol drinking status, occupation noise exposure, and cardiovascular diseases. Detailed classifications were presented in Supplementary Methods.

#### 2.1.4. Statistics Analysis

We first analyzed the association between DM (total DM, T1D, and T2D) and SNHL (total, unilateral and bilateral SNHL), followed by DM classification by HbA1c levels, and bilateral SNHL classification by insufficient and poor severity levels. Besides, we analyzed these relationships between DM and SNHL in subgroups stratified by sex (female or male) and age (< 60 or ≥ 60 years old).

Multivariate logistic regression analyses were conducted to examine the cross‐sectional association between diabetes and SNHL. Model adjusted for age, sex, BMI, ethnic background, education, employment status, smoking status, alcohol drinking status, duration of noisy workplace exposure, and cardiovascular diseases was applied to minimize the impact of potential confounding factors.

All statistical analyses were performed using R3.6.3 software and *p* value of < 0.05 was considered statistically significant.

### 2.2. Shared Genetic Architecture Between DM and SNHL

In this second part, we further explored their shared genetic architecture and elucidated potential underlying biological mechanisms.

#### 2.2.1. GWAS Resources and Meta‐Analysis

Based on the phenotypic and genotyping data from the UK Biobank population, we conducted a GWAS analysis for SNHL. In the observational analysis, SNHL was primarily defined using SRT‐based criteria, whereas in the GWAS analysis, a broader definition was adopted by additionally incorporating self‐report, hearing aid use, and ICD‐coded cases to increase sample size and statistical power. We also obtained GWAS data for T1D and T2D. The detailed procedures were provided in the Supplementary Methods and Table S2.

#### 2.2.2. Global and Local Genetic Correlation Analyses of DM and SNHL

In a GWAS, the effect size estimate for each SNP is influenced by linkage disequilibrium (LD) with other SNPs. To estimate the global genetic correlation between T1D, T2D, and SNHL, we applied LD Score Regression (LDSC). The Rg value ranges from −1 (indicating complete negative correlation) to 1 (indicating complete positive correlation) [30, 31]. LD score estimation was performed using HapMap3 SNPs obtained from the 1000 Genomes Project (Phase 3 EUR).

Stratified LDSC was used to better understand the heritability enrichment similarities between T1D, T2D, and SNHL at the functional category level across 28 functional categories, with details in the Supplementary Methods. A false discovery rate (FDR) threshold of 0.05/28 = 0.00178 was applied to define significant enrichment.

#### 2.2.3. Genetic Causality Inference Analysis of T2D and SNHL

We conducted a comprehensive bidirectional two‐sample MR analysis to identify potential causal relationships, using five methods: inverse variance weighted (IVW), MR‐Egger, simple mode, weighted mode, and weighted median. To ensure robustness, the instrumental variables (IVs) in our MR analysis must satisfy three core assumptions (Supplementary Methods). Meanwhile, IVs meeting the following criteria were excluded: (1) IVs missing from the GWAS data of the outcomes and (2) palindromic IVs with intermediate allele frequencies (0.42–0.58).

Additionally, we employed three sensitivity analysis approaches: Cochran’s Q heterogeneity test, MR‐Egger pleiotropy test, and MR Pleiotropy Residual Sum and Outlier (MR‐PRESSO) pleiotropy test, to verify heterogeneity and pleiotropy of IVs. IVs that were considered to be outliers or associated with the outcome (*p* < 0.05) were excluded in the second round of MR to minimize the heterogeneity and pleiotropy.

Considering the overlap of UK Biobank samples between T2D GWAS study (Xue A et al.) and the SNHL GWAS study, we used only the T2D GWAS summary statistics from FinnGen for a bidirectional causal effect MR analysis between T2D and SNHL, in order to minimize false positive results [32].

The statistical analysis process for MR was conducted using the TwoSampleMR package in R version 3.6.3. For all MR analyses, we maintained a significance threshold of *p* < 0.05 for individual tests.

#### 2.2.4. Identification of Pleiotropic Loci for SNHL‐T2D by Cross‐Trait Meta‐Analysis

A joint analysis strategy using both multi‐trait analysis of GWAS (MTAG) and cross‐phenotype association studies (CPASSOC) was applied to identify pleiotropic loci between diabetes and SNHL. *p*‐Value thresholds of both P_MTAG_ and P_CPASSOC_ < 5 × 10^−8^ were considered to satisfy the criteria for the pleiotropic SNP and locus.

Functional mapping and annotation of GWAS (FUMA, https://fuma.ctglab.nl, version 1.5.4) was used to perform clumping on the cross‐trait GWAS summary data. The analysis was based on the 1000 Genomes Project (Phase 3 EUR) data, with an r^2^ threshold of 0.6 employed to distinguish independent loci, and an r^2^ threshold of 0.1 used to identify lead SNPs.

Based on reference data from the 1000 Genomes Project (Phase 3 EUR), fine‐mapping utilized the LD structure between SNPs to calculate the posterior probability of candidate SNPs being causal variants. This approach was used to validate whether the lead SNPs inferred by MTAG were credible shared variants.

We further obtained data from the IEU OpenGWAS project (https://gwas.mrcieu.ac.uk/phewas/) [33] to perform a phenome‐wide association study (PheWAS) of the shared loci. PheWAS results were filtered using a significance threshold of *p*‐values less than 5 × 10^−8^.

#### 2.2.5. Gene Annotation and Priority for SNHL‐T2D

Five methods were applied to jointly infer the protein‐coding genes associated with the lead SNPs of HL‐T2D identified by MTAG and CPASSOC: (1) nearest location, (2) expression quantitative trait loci (eQTL) colocalization, (3) high‐throughput chromatin conformation capture (c) colocalization, (4) multi‐marker analysis of genomic annotation (MAGMA), and (5) summary‐based Mendelian randomization (SMR). The criteria for gene prioritization were detailed in the Supplementary Methods.

Genes with evidence from at least three of the following five methods were prioritized as credible genes for the SNHL‐T2D trait pair.

#### 2.2.6. Functional Annotation for SNHL‐T2D

The summary data for the T2D‐SNHL trait pair by MTAG and CPASSOC were uploaded to website of FUMA (v1.5.3) [34] for visualization and genome‐wide gene‐set enrichment analyses. Pathway analysis for gene sets was conducted by MAGMA through gene ontology (GO) gene sets from the Molecular Signatures Database (MSigDB, v6.2) [35]. GO biological pathways with *p* < 0.05/*n* (where *n* = 10,525 for MTAG and *n* = 10,529 for CPASSOC) were considered to be significantly associated with the shared traits, while those with *p* < 1/*n* were considered to be moderately associated.

We further performed over‐representation pathway analysis using WebGestalt online tool (https://www.webgestalt.org) [36] to identify the biological processes in which shared genes were involved in the co‐occurrence of SNHL and T2D.

#### 2.2.7. Tissue and Cell‐Type Specific Enrichment of SNHL‐T2D

Tissue‐enrichment analyses for the MTAG data and the shared genes were carried out within 54 tissues from the GTEx (v8) on FUMA to identify the tissues where shared heritability were enriched and to provide insights into potential tissue locations for therapeutic intervention [37].

Given tissues directly related to hearing were not included in the GTEx database, we further explored the cell type enrichment of the identified shared genes in cochlear cell clusters based on single‐cell transcriptomic (scRNA) data, which classified the cochlea of 15‐day‐old mice into inner hair cells (IHCs), outer hair cells (OHCs), and Deiters’ cells [38].

#### 2.2.8. Exploration for Therapeutic Targets for T2D‐SNHL

In this study, we performed gene‐target drug analysis using the DGIdb (Drug–Gene Interaction Database). The DGIdb database (Version: 20240410, https://www.dgidb.org/) includes comprehensive data on over 40,000 genes and 10,000 drugs, documenting more than 100,000 drug–gene interactions and providing details on the approval status of these drugs [39–41]. This resource enables us to assess the druggability of the genes of interest by utilizing its drug–gene interaction data, and to identify existing drugs that interact with the target genes, which could serve as potential targeted therapeutic options [42].

## 3. Result

**Figure 1:**
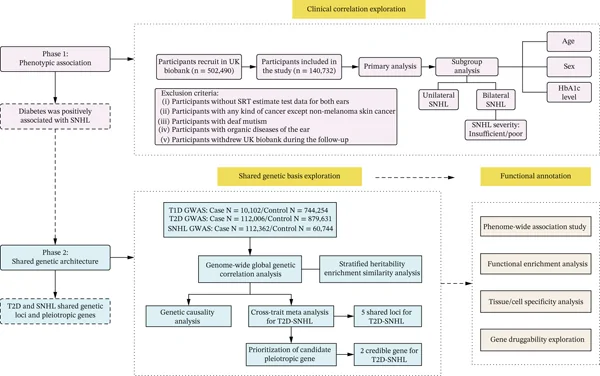
Study design and analysis workflow.

The results of the whole study were divided into two main parts, including the clinical association and genetic correlation between DM and SNHL, as shown in the flowchart (Figure 1).

### 3.1. Phenotypic Association Between DM and SNHL

#### 3.1.1. Baseline Characteristics of Study Participants

Our study included 502,490 participants from the UK Biobank. After exclusions, 140,732 participants remained, consisting of 57,190 cases of SNHL, including 17,531 bilateral SNHL cases and 39,659 unilateral SNHL cases. Additionally, 11,557 participants had DM, with 1200 diagnosed with T1D and 10,869 with T2D (Figure 1, Table S2).

Detailed baseline characteristics are presented in Tables S3, S4 and S5. Specifically, compared with the controls, participants diagnosed with SNHL were more likely to be male, older, obese, retired, and worked in noisy place for more than 5 years. Additionally, SNHL participants exhibited a higher prevalence of hypertension, cardiovascular diseases, and hyperlipidemia. Furthermore, they were more likely to report the use of ototoxic medications.

#### 3.1.2. Epidemiological Association Between DM and SNHL

**Figure 2:**
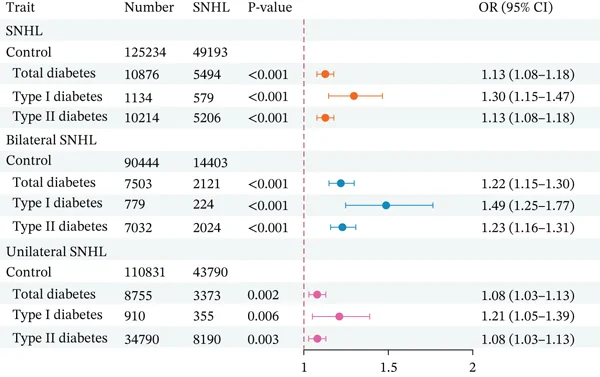
Phenotypic association between DM and SNHL. *Note:* Bold font indicates significance at *p* < 0.05. Statistic method: multivariable logistic regression.

After adjusting for age, sex, BMI, ethnic background, education, employment status, smoking status, alcohol drinking status, duration of noisy workplace exposure, and cardiovascular diseases, DM was found to be significantly associated with SNHL, including both bilateral and unilateral SNHL (total SNHL: OR 1.13, 95% CI 1.08–1.18, *p* < 0.001; bilateral SNHL: OR 1.22, 95% CI 1.15–1.3, *p* < 0.001; unilateral SNHL: OR 1.08, 95% CI 1.03–1.13, *p* = 0.002). Both T1D and T2D revealed significant associations between total, bilateral, and unilateral SNHL (Figure 2, Table 1).

**Table 1:** Multivariable analysis for SNHL in DM participants.

Exposure	Number	SNHL	OR (95% CI)	*p*‐Value	Number	Bilateral SNHL	OR (95% CI)	*p*‐Value	Number	Unilateral SNHL	OR (95% CI)	*p*‐Value
Control	125234	49193	Reference		90444	14403	Reference		110831	34790	Reference	
Total diabetes	10876	5494	1.13 (1.08–1.18)	**< 0.001**	7503	2121	1.22 (1.15–1.3)	< 0.001	8755	3373	1.08 (1.03–1.13)	0.002
T1D	1134	579	1.3 (1.15–1.47)	**< 0.001**	779	224	1.49 (1.25–1.77)	< 0.001	910	355	1.21 (1.05–1.39)	0.008
T2D	10214	5206	1.13 (1.08–1.18)	**< 0.001**	7032	2024	1.23 (1.16–1.31)	< 0.001	34790	8190	1.08 (1.03–1.13)	0.003

*Note:* Model: Adjusted for age, sex, BMI, ethnic background, education, employment status, smoking status, alcohol drinking status, time of working in noisy place, cardiovascular disease (including coronary atherosclerosis, angina pectoris, myocardial infarction, ischemic stroke, hemorrhagic stroke). Bold *p* values indicate statistical significance at *p* < 0.05.

Abbreviations: DM, diabetes mellitus; SNHL, sensorineural hearing loss; T1D, type 1 diabetes; T2D, type 2 diabetes.

We subsequently investigated the relationships between DM with various HbA1c levels and SNHL, compared with those without DM. Results indicated significant positive associations between DM with mildly elevated and high HbA1c levels and SNHL (mildly elevated HbA1c: OR 1.123, 95% CI 1.05–1.201, *p* < 0.001; high HbA1c: OR 1.167, 95% CI 1.092–1.247, *p* < 0.001). Furthermore, elevated HbA1c levels and a high prevalence of T2D were significantly positively associated with SNHL. However, in patients with T1D, even HbA1c levels within the normal range (31–< 39 mmol/mol) were associated with a higher risk of SNHL, indicating the potential involvement of non‐glycemic mechanisms in the development of SNHL in patients with T1D (Table S6). Moreover, bilateral SNHL was classified into insufficient and poor degrees, and patients with all types of DM exhibited a significantly higher prevalence of both insufficient and poor levels of HL (Table S7).

#### 3.1.3. Epidemiological Associations Between DM and SNHL in Sex and Age Subgroups

To further investigate the potential mediating effects of sex and age, we then performed a subgroup analysis of DM and SNHL by sex (female or male) and age (< 60 years or ≥ 60 years). Both male (OR 1.17, 95% CI 1.11–1.24, *p* < 0.001) and female (OR 1.16, 95% CI 1.09–1.24, *p* < 0.001) patients diagnosed with DM showed a consistent risk of SNHL (p − interaction = 0.394) (Table S8). Besides, in the age‐stratified multivariable analysis, all patients with DM under 60 years of age (< 60 years: OR 1.21, 95% CI 1.13–1.29, *p* < 0.001) and those aged 60 years or older (≥ 60 years: OR 1.21, 95% CI 1.13–1.29, *p* < 0.001) had a significantly increased risk of any type of SNHL, with no significant difference observed between the age subgroups (p − interaction = 0.530) (Figure S1, Table S8).

We further analyzed the associations between DM and SNHL subtypes within the sex‐specific subgroups. Among male and female patients with all types of DM (total DM, T1D, T2D), significant associations were observed with bilateral SNHL (Table S9). However, in the T1D subgroup, female patients showed no significant association between T1D and unilateral SNHL, whereas male individuals with T1D had a significantly higher risk of unilateral SNHL. Meanwhile, for patients with T2D, the risk of both bilateral and unilateral SNHL was consistent and similar in both male and female groups (Table S9).

### 3.2. Shared Genetic Architecture Between DM and SNHL

#### 3.2.1. Genetic Correlation and Causality Between DM and SNHL

Given the observed phenotypic association between T2D and SNHL, we further calculated global and stratified LDSC based on GWAS summary data to characterize the genetic correlation between DM and SNHL. LDSC revealed that SNHL exhibited a positive genetic correlation with T2D (Rg = 0.0982, *p* = 0.0095), but not with T1D, suggesting shared genetic basis between SNHL and T2D, as shown in Table S10.

**Figure 3:**
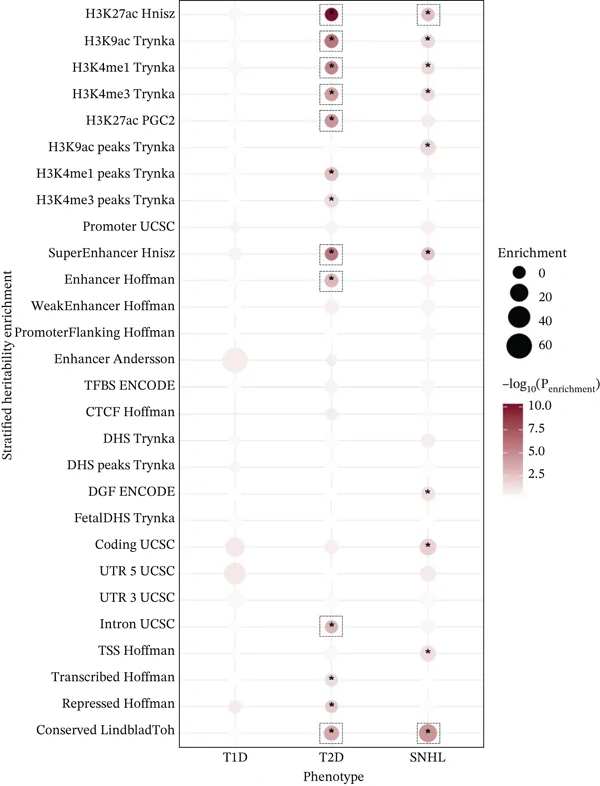
Stratified heritability enrichment functional regions for DM and SNHL. *Note:* T1d, type I diabetes; T2D, type II diabetes; SNHL, sensorineural hearing loss; The box indicates P_Bonferroni_ < 0.00178 (0.05/28);  ^∗^ indicates *p* < 0.05.

Stratified LDSC further exhibited genetic similarity in local functional regions between T2D and SNHL, with shared enrichment in conserved regions, chromatin modification mark regions, and super‐enhancer regions. However, the heritability enrichment regions of T1D were observed to differ from those of T2D and SNHL, with no significant enrichment found in these functional genetic partitions (Figure 3 and Tables S11, S12 and S13).

Bidirectional two‐sample MR was performed to clarify whether the observed association was causal or a result of shared genetic foundations. After excluding outcome‐related IVs and outliers (T2D: from 187 IVs to 169 IVs; SNHL: from 7 IVs to 6 IVs) (Table S14 and Table S15), the IVs in the second round of forward and reverse MR analysis exhibited minimal heterogeneity and pleiotropy (Table S16). However, among the five methods applied, only the IVW approach suggested a potential positive causal relationship from T2D to SNHL (b = 0.02, *p* = 0.05), whereas the other methods did not support a consistent forward or reverse causal relationship between T2D and SNHL (Table S16).

#### 3.2.2. Identification of Shared Loci for T2D‐SNHL

Given the shared genetic basis identified between T2D and SNHL, we applied both MTAG and CPASSOC to identify the cross‐trait loci between them (Figure S2, Figure S3). After clumping, we identified 11 genome‐wide LD‐independent significant variants (GWAS, p < 5 × 10^−8^, r^2^ < 0.6), represented by six lead variants (r^2^ < 0.1). Risk loci were defined by including all lead variants and merging loci within 250 kb. This resulted in five independent risk loci for SNHL‐T2D. Among these, four loci with representative lead SNP (rs6453022, rs56299331, rs10901863, and rs36062310, respectively) satisfying both P_MTAG_ < 5 × 10^−8^ and P_CPASSOC_ < 5 × 10^−8^ were retained as credible loci for subsequent analyses for T2D‐SNHL, whereas one locus represented by rs351238 was not retained because its P_CPASSOC_ did not meet the significance threshold. Among the lead SNPs of these four loci, rs56299331 have not been previously reported to be associated with T2D or SNHL in previous researches (Table 2).

**Table 2:** Cross‐trait meta‐analysis results between SNHL and T2D.

Locus	Previously published (PMID)	Lead SNP	GRCh37	RA	NRA	Nearest gene	EQTL genes	Hi‐C genes	SMR gene	MAGMA gene	P (MTAG)	P (CPASSOC)	Credible shared locus
1	34108613	rs6453022	5:73076511	C	A	*ARHGEF28*	ARHGEF28	*ARHGEF28*	*ARHGEF28*	*ARHGEF28*	**7.51E−16**	**5.46E−24**	**Yes**
							ANKRA2						
2	Novel	rs56299331	10:114788436	T	C	*TCF7L2*	TCF7L2	*TECTB*	NA	*TCF7L2*	**4.35E−15**	**2.76E−319**	**Yes**
								*ZDHHC6*					
								*VTI1A*					
3	32986727	rs10901863	10:126812270	T	C	*CTBP2*	NA	NA	NA	NA	**4.79E−09**	**5.88E−29**	**Yes**
	34108613												
	35661827												
	34594039												
4	32986727	rs36062310	22:50988105	A	G	*KLHDC7B*	*CHKB*	NA	*TYMP*	NA	**9.27E−09**	**2.36E−09**	**Yes**
	34108613												
	35661827												
5	Novel	rs351238	15:74477216	A	G	*STRA6*	*ISLR2*	NA	*ULK3*	NA	**4.23E−08**	1.73E−02	No
							*ISLR*						

*Note:* Novel locus indicates that it has not been previously reported to be associated with T2D or SNHL in previous published researches. Bold font indicates significance at p <5 × 10‐8. Credible shared loci were defined as loci whose representative lead SNPs satisfied both P_MTAG_<5 × 10^-8^ and P_CPASSOC_<5 × 10^-8^. Bold *p* values indicate statistical significance at *p* < 5 × 10^-8^.

Abbreviations: CPASSOC, cross‐phenotype association study; eQTL, expression quantitative trait loci; GRCh37, Genome Reference Consortium Human genome build 37; Hi‐C, high‐throughput chromosome conformation capture; MTAG, multitrait analysis of GWAS; NRA, non‐risk allele; PMID, PubMed ID; RA, risk allele; SMR, summary‐based Mendelian randomization; SNHL, sensorineural hearing loss; SNP, single nucleotide polymorphism; T2D, type 2 diabetes.

Fine‐mapping for these four lead SNPs revealed that two variants were mapped with high causal probabilities in the 99% credible set (with cumulative posterior probabilities greater than 0.8 for both traits) and one variant were within the high posterior probability for the single trait. Most SNPs were mapped within the 99% credible set, further strengthening the robustness of the results (Table S17).

To identify the phenotypes associated with shared SNPs, we searched the PheWAS results for each SNP. Phenotype association results revealed that rs6453022 and rs10901863 were significantly associated with SNHL‐related traits, rs36062310 was associated with SNHL and red blood cell indices, and rs56299331 was associated with T2D and glucose levels (Table S18).

#### 3.2.3. Functional Annotation and Priority for Pleiotropic Gene for T2D‐SNHL

A total of 14 genes were identified as being associated with four lead SNPs as meeting at least one of the gene prioritization criteria (Method). Among these, *ARHGEF28* (as the nearest gene, eQTL gene, Hi‐C gene, SMR gene, and MAGMA gene in locus 1) and *TCF7L2* (as the nearest gene, eQTL gene, and Hi‐C gene for locus 2) satisfied at least three of all five prioritization criteria and were considered credible shared genes for T2D and SNHL (Table 2, Tables S19, S20, S21, S22 and S23 and Figure S4).

#### 3.2.4. Identification of Biological Processes Involved in the Shared Genetic Mechanisms for T2D‐SNHL

To identify the enrichment characteristics of shared genetic signals in specific tissues, we performed MAGMA enrichment analysis utilizing GO dataset for functional annotation. Pathways related to glucose metabolism and organogenesis, such as regulation of pancreatic B‐cell apoptosis, insulin binding, cytoplasmic stress granules, and regulation of biosynthetic metabolism (*p* < Bonferroni *p*), as well as cochlear morphogenesis and pancreas development (*p* < 1/*n*), were enriched (Table S24, Table S25). Additionally, genes shared between T2D and SNHL were mainly enriched in biological processes of ephrin receptor signaling and pancreas development (*p* < 0.05) on WebGestalt (Table S26).

#### 3.2.5. Tissue and Cell Specificity Analysis for T2D‐SNHL

To identify specific tissues in which the enrichment of shared heritability were enriched, we tested the enrichment signals in the GTEx dataset across 54 tissues. We observed prominent enrichment in some reproductive system tissues, including cervix, ovary, and testis, and moderate enrichment signals in other reproductive tissues such as the uterus and fallopian tubes, as well as in the cerebellum (Figure S5). Additionally, tissue enrichment analysis of shared genes also highlighted signals in the reproductive system and further emphasized the role of adipose and vascular tissues in the pathogenesis of T2D and SNHL (Figure S6).

Meanwhile, the cochlear scRNA data showed that *ARHGEF28* were significantly enriched in OHCs, compared with other cells (IHCs and Deiters cells), whereas no enrichment of shared genes was observed in IHCs or Deiters’ cells, indicating that OHCs might play a pathogenic role in SNHL.

#### 3.2.6. Druggability Assessment of Shared Genes for T2D‐SNHL

To identify candidate drugs for the two shared genes for T2D‐SNHL, we searched the DGIdb dataset for drug–gene interaction information. Data from a drug database (Pharmacogenomics Knowledge Base, PharmGKB) indicated that *ARHGEF28* was linked to methylphenidate hydrochloride, a central nervous system stimulant, while *TCF7L2* was associated with a broader range of drugs, including antidiabetic agents (e.g., DPP‐4 inhibitors, metformin, and repaglinide), immunosuppressants (e.g., cyclosporine and sirolimus), antibiotics (e.g., sulfonamides), and diuretics (e.g., hydrochlorothiazide) (Table 3).

**Table 3:** Gene‐drug interactions of shared genes in the DGIdb database.

Gene	Source of database	Database version	Drug	Identity	Clinical phase
ARHGEF28	PharmGKB	2004/5/24	Methylphenidate hydrochloride	Central nervous system stimulant	Approved
TCF7L2	PharmGKB	2004/5/24	Metformin	Antidiabetic agent	Approved
TCF7L2	PharmGKB	2004/5/24	Dipeptidyl peptidase 4 (DPP‐4) inhibitors	Antidiabetic agent	Approved
TCF7L2	PharmGKB	2004/5/24	Repaglinide	Antidiabetic agent	Approved
TCF7L2	PharmGKB	2004/5/24	Cyclosporine	Immunosuppressant	Approved
TCF7L2	PharmGKB	2004/5/24	Sirolimus	Immunosuppressant	Approved
TCF7L2	PharmGKB	2004/5/24	Sulfonamides	Antibiotic	Approved
TCF7L2	PharmGKB	2004/5/24	Hydrochlorothiazide	Diuretic	Approved

*Note:* DGIdb version: 10‐April‐2024.

Abbreviations: DGIdb, Drug–Gene Interaction Database; PharmGKB: Pharmacogenetics and Pharmacogenomics Knowledge Base.

## 4. Discussion

In this study, we observed consistent epidemiological associations between DM and SNHL in a large UK Biobank cohort and further identified a shared genetic basis between T2D and SNHL. Unlike some phenotypic association analysis [12, 16–18], MR analysis did not provide consistent evidence for a causal relationship between T2D and SNHL, supporting the hypothesis that their comorbidity was partially driven by a shared genetic basis. Together, these findings extend current understanding of the relationship between T2D and SNHL and provide a framework for interpreting their shared biology.

Consistent with previous studies, both T1D and T2D were significantly associated with SNHL [43]. Moreover, among patients with T1D and T2D with varying glycated hemoglobin levels, we observed a significant association with SNHL not only in the high glycated hemoglobin group (≥ 48 mmol/mol) [44] but also in the elevated glycated hemoglobin group (prediabetes, 31~<39 mmol/mol), indicating a consistent risk across different glycemic statuses. Additionally, diabetes was linked to various types of hearing impairment, including both unilateral and bilateral SNHL, as well as mild and severe hearing loss. These findings underscore the widespread association and comorbidity potential between diabetes and SNHL, highlighting the need for hearing health assessment and intervention in diabetes management [12]. Interestingly, we found that in patients with T1D, even HbA1c levels within the normal range were associated with a higher risk of SNHL, suggesting the involvement of T1D‐specific immune‐related pathological mechanisms. In contrast, the overall genetic correlation observed between T2D and SNHL, which is absent in T1D, likely reflects shared metabolic and vascular‐related genetic mechanisms rather than the immune‐related mechanisms underlying SNHL in patients with T1D [45]..

Interestingly, tissue enrichment analysis revealed significant signals in reproductive tissues and moderate enrichment in the cerebellum. These findings do not necessarily indicate direct organ‐specific involvement in the pathogenesis of T2D with comorbid SNHL (T2D‐SNHL). Rather, they may reflect shared regulatory architecture and broader endocrine and metabolic programs. Given the absence of inner ear tissues in the GTEx resource, these signals may only indirectly capture biological features relevant to auditory dysfunction. In contrast, the enrichment of *ARHGEF28* in OHC identified from cochlear single‐cell data may provide a more disease‐relevant clue to the shared genetic basis of T2D and SNHL.

We identified several approved drugs interacting with the shared genes for T2D‐SNHL, with metformin being the most notable candidate. Metformin, a first‐line antidiabetic agent, is known to improve insulin sensitivity and reduce inflammation in T2D [46]. Additionally, it exerts neuroprotective effects that may lower the risk of SNHL [47, 48], and this SNHL‐protective effect has also been validated in patients with T2D [49]. Our findings suggest that metformin may represent a candidate therapeutic direction for T2D‐SNHL comorbidity, although further functional and clinical validation is needed. DPP‐4 inhibitors, such as sitagliptin, enhance incretin hormone action, thereby improving glucose homeostasis and potentially reducing hyperglycemia‐induced oxidative stress [50], a key mechanism in SNHL. Repaglinide, a non‐sulfonylurea (meglitinide) insulin secretagogue, is primarily used for postprandial blood glucose control in diabetes [51], and no risk of hearing loss has been identified with its use. Besides, cyclosporine [52] and sirolimus [53, 54] have been found to potentially have therapeutic effects on SNHL, but their role in the treatment of T2D remains unclear. Therefore, DPP‐4 inhibitors, repaglinide, cyclosporine, and sirolimus may hold therapeutic potential for T2D‐SNHL comorbidity, but further research is needed to confirm their efficacy and underlying mechanisms. Among the other drugs, previous studies have suggested that methylphenidate, a medication for attention‐deficit hyperactivity disorder (ADHD), might lead hearing loss as side effect [55, 56]. Therefore, the use of methylphenidate should be minimized in individuals at risk for or diagnosed with SNHL or SNHL‐T2D comorbidity.

This study has several limitations. First, all data analyzed in this study were derived from individuals of European ancestry, which might limit the generalizability of our findings to other populations. Future studies including more diverse ancestral groups are needed to validate the robustness of these results. Second, the incomplete capture of ICD codes exists for certain UK Biobank participants, which may be attributed to earlier medical consultations or healthcare visits occurring outside the UK Biobank data collection framework. As a result, some diagnoses may not have been recorded within the available hospital episode statistics. To maximize the identification of SNHL, T2D and T1D cases, we integrated both ICD codes and self‐reported data. Although this approach improves case ascertainment, it may also introduce potential false positives and misclassification bias, because self‐reported diagnoses may be inaccurate, incomplete, or not fully consistent with clinical coding records. Third, the definition of SNHL was not entirely identical between the observational and GWAS analyses. The observational analysis relied primarily on SRT‐based criteria, whereas the GWAS definition additionally incorporated self‐report, hearing aid use, and ICD‐coded cases. Although this broader definition may improve sample size and statistical power, it may also increase phenotypic heterogeneity and should be considered when interpreting the genetic correlation and shared locus analyses. Additionally, though MR results indicate that the observed association and genetic overlap is more likely due to pleiotropy rather than causality, further studies with larger sample sizes are needed to confirm these findings. Finally, although this study indicated a significant genetic correlation between T2D and SNHL, the Rg value and *p*‐value were not particularly strong, which might limit the number of identified shared loci and genes, and increase the likelihood of false positives. Therefore, this study employed a combination method of MTAG and CPASSOC to identify shared loci and used five gene prioritization methods to refine gene selection, thereby improving the accuracy of gene identification.

## 5. Conclusion

These findings progress our understanding of the epidemiological association, shared genetic pathogenesis and potential therapeutic targets between T2D and SNHL in European‐ancestry populations. These findings emphasize the need for early screening and hearing function assessment in patients with DM, and future research should focus on functional validation of candidate drugs for targeted therapies addressing both metabolic and auditory dysfunctions.

**Nomenclature**
BMI: body mass index
CPASSOC: cross‐phenotype association studies
DGIdb: Drug–Gene Interaction Database
DM: diabetes mellitus
eQTL: expression quantitative trait loci
FDR: false discovery rate
FUMA: functional mapping and annotation of GWAS
GO: gene ontology
GWAS: genome‐wide association study
IHC: inner hair cells
IV: instrumental variable
IVW: inverse‐variance weighted
LD: linkage disequilibrium
LDSC: linkage disequilibrium score regression
MAGMA: multi‐marker analysis of genomic annotation
MR‐PRESSO: Mendelian Randomization Pleiotropy Residual Sum and Outlier
MTAG: multi‐trait analysis of GWAS
NHANES: National Health and Nutrition Examination Survey
OHC: outer hair cells
PharmGKB: Pharmacogenomics Knowledge Base
PheWAS: phenome‐wide association study
scRNA: single‐cell RNA sequencing
SE: standard error
SMR: summary‐data‐based Mendelian randomization
SNHL: sensorineural hearing loss
SRT: speech‐reception‐threshold
T1D: type 1 diabetes mellitus
T2D: type 2 diabetes mellitus

## Author Contributions

X.W., Z.Z., and K.Q. wrote the main manuscript text and prepared all the tables and figures. X.W. also contributed to study design and data acquisition. Y.L. and X.Y. developed the data analysis methodology and performed formal analyses. W.P. was responsible for funding acquisition. J.R., D.J., and Y.Z. contributed to resource acquisition, project administration, supervision, and manuscript revision. Y.Z. also contributed to the funding acquisition had primary responsibility for the overall study. X.W., Z.Z., and K.Q. authors contributed equally to this article as co‐first authors.

## Funding

This study was supported by West China Hospital, Sichuan University (ZY, grant #ZYJC21027; PWD, grant: 2023HXBH119); Chengdu Science and Technology Bureau (ZY, grant #2022‐YF05‐01387‐ SN); The Science and Technology Department of Sichuan Province (ZY, grant #2024YFHZ0076; PWD, grant #2025ZNSFSC1526); The Foundation of National Clinical Research Center for Geriatrics (ZY, grant #Z2023LC002); National Natural Youth Science Foundation of China (PWD, grant #82301307); Chengdu Science and Technology Bureau (ZY, grant #2024‐YF05‐00050‐SN); Health Care Research Project of Sichuan Province (ZY, grant #ZH2025‐102); National Key Research and Development Program of China (ZY, grant #2024YFC2418305),

## Disclosure

All authors reviewed and approved the final manuscript.

## Ethics Statement

The data for this study were sourced from the UK Biobank, which received approval from the National Health Service National Research Ethics Service (ref. 21/NW/0157) and all the analyses conducted in this study were approved by UK Biobank research committee (Application number: 69718, 80787).

## Consent

The authors have nothing to report.

## Conflicts of Interest

The authors declare no conflicts of interest.

## Supporting Information

Additional supporting information can be found online in the Supporting Information section.

## Supporting information


**Supporting Information 1** **STROBE-MR checklist:** Completed STROBE‐MR checklist for reporting Mendelian randomization studies, based on the STROBE‐MR Statement (doi:10.1001/jama.2021.18236).

**Supporting Information 2** Supplementary Method. 1. Phenotypic association analyses between DM and SNHL. 1.1. Definition of DM and SNHL. 1.2. Information of covariate. 2. Shared genetic architecture between DM and SNHL. 2.1. GWAS resources and meta‐analysis. 2.2. Global and local genetic correlation analyses of DM and SNHL. 2.3. Genetic causality inference analysis of T2D and SNHL. 2.4. Identification of pleiotropic loci for SNHL‐T2D by cross‐trait meta‐analysis. 2.5. Gene annotation and priority for SNHL‐T2D.

**Supporting Information 3** Figure S1 Phenotypic association between DM and SNHL across age and sex subgroups. Figure S2 Manhattan plot of summary results from cross‐trait meta‐analyses of T2D and SNHL. Figure S3 QQ plot of summary results from cross‐trait meta‐analyses of T2D and SNHL. Figure S4 MAGMA genes of summary results from cross‐trait meta‐analyses of T2D and SNHL. Figure S5 Enrichment results of cross‐trait meta‐analyses of T2D‐SNHL in 54 GTEx tissues. Figure S6 Enrichment of shared genes for T2D‐SNHL across 54 GTEx tissues.

**Supporting Information 4** Table S1 Exclusion Criteria for the Cross‐Sectional Study. Table S2 All GWAS data sources in the study. Table S3 Clinical and demographic characteristics for total SNHL study. Table S4 Clinical and demographic characteristics for bilateral SNHL study. Table S5 Clinical and demographic characteristics for unilateral SNHL study. Table S6 Association between HbA1c groups and SNHL in diabetes patients. Table S7 Association between diabetes and severity of bilateral SNHL. Table S8 Association between diabetes and SNHL across sex and age subgroups. Table S9 Cross‐association of different diabetes types with varying degree of bilateral SNHL across age and sex subgroups. Table S10 Genetic correlations in SNHL and diabetes. Table S11 Stratified heritability enrichment results for T1D. Table S12 Stratified heritability enrichment results for T2D. Table S13 Stratified heritability enrichment results for SNHL. Table S14 Instrumental variable information for sensorineural hearing loss. Table S15 Instrumental variable information for type 2 diabetes. Table S16 Bidirectional MR analysis of T2D and SNHL. Table S17 Fine‐mapping results for shared loci Table S18 Phenome‐wide association analysis results for shared loci Table S19 eQTL genes associated with lead SNPs in MTAG in diabetes‐related tissues. Table S20 Gene mapping of shared loci using Hi‐C interaction in diabetes‐related tissue. Table S21 Summary‐based Mendelian Randomization (SMR) analysis of shared loci to identify expression quantitative trait loci (eQTL) genes with causal effects Table S22 Gene association results from MTAG using MAGMA. Table S23 Gene association results from CPASSOC using MAGMA (top 100). Table S24 GO enrichment for MTAG in MAGMA. Table S25 GO enrichment for CPASSOC in MAGMA. Table S26 GO enrichment for identified shared genes

## Data Availability

The relevant data for the observational study from the UK Biobank cohort can be accessed with permission from the UK Biobank (https://www.ukbiobank.ac.uk/enable-your-research/apply-for-access). Genetic data for T1D and T2D were obtained from FinnGen (https://www.finngen.fi/en/access_results), the study by Xue et al. (doi:10.1038/s41467-018-04951-w; available via the IEU OpenGWAS project: https://gwas.mrcieu.ac.uk/datasets/ebi-a-GCST006867/), and the study by Joshua Chiou et al. (doi:10.1038/s41586-021-03552-w; accessible through the GWAS Catalog: https://www.ebi.ac.uk/gwas/studies/GCST90014023).
