# Supplementary material for: Conventional and Shared Genetic Association Analysis Between Diabetes Mellitus and Sensorineural Hearing Loss
Source: Hum Mutat. 2026 Sep 15;2026:5099141. doi: 10.1155/humu/5099141 (PMC13576905; doi:10.1155/humu/5099141)
Supplement: Supplementary file 1 [file HUMU-2026-5099141-s002.docx]

**STROBE-MR checklist of recommended items to address in reports of Mendelian randomization studies**^1^ ^2^

| **Item No.** | **Section** | **Checklist item** | **Page No.** | **Relevant text from manuscript** |
| --- | --- | --- | --- | --- |
| 1 | **TITLE and ABSTRACT** | Indicate Mendelian randomization (MR) as the study’s design in the title and/or the abstract if that is a main purpose of the study | 3 | Abstract: Genetic correlations and causality were examined by linkage disequilibrium score regression and bidirectional Mendelian randomization. |
|  | **INTRODUCTION** |  |  |  |
| 2 | **Background** | Explain the scientific background and rationale for the reported study. What is the exposure? Is a potential causal relationship between exposure and outcome plausible? Justify why MR is a helpful method to address the study question | 5 | Although prior researches have reported on the association between DM and SNHL[1-4], Mendelian randomization (MR) analysis for causality inference between DM and SNHL is still lacking |
| 3 | **Objectives** | State specific objectives clearly, including pre-specified causal hypotheses (if any). State that MR is a method that, under specific assumptions, intends to estimate causal effects | 5 | Although prior researches have reported on the association between DM and SNHL[1-4], Mendelian randomization (MR) analysis for causality inference between DM and SNHL is still lacking |
|  | **METHODS** |  |  |  |
| 4 | **Study design and data sources** | Present key elements of the study design early in the article. Consider including a table listing sources of data for all phases of the study. For each data source contributing to the analysis, describe the following: |  |  |
|  | a) | Setting: Describe the study design and the underlying population, if possible. Describe the setting, locations, and relevant dates, including periods of recruitment, exposure, follow-up, and data collection, when available. | 8 | Based on the phenotypic and genotyping data from the UK Biobank population, we conducted a GWAS analysis for SNHL. We also obtained GWAS data for T1D and T2D. The detailed procedures were provided in the Supplementary Methods and Table S2. |
|  | b) | Participants: Give the eligibility criteria, and the sources and methods of selection of participants. Report the sample size, and whether any power or sample size calculations were carried out prior to the main analysis | 4,5 (Supplementary Methods) | See Supplementary Methods:  For SNHL, to enhance the statistical power of GWAS and improve the detection of rare variants, we expanded the definition of SNHL in the GWAS analysis. In addition to selecting SNHL participants based on an SRT threshold of -5.5 (Section 2.1.3), other participants meeting at least one of the following criteria were also included as SNHL cases: (1) answered 'yes' to 'Hearing difficulty/problems' (Data Field 2247); (2) answered 'yes' to 'Hearing difficulty/problems with background noise' (Data Field 2257); (3) answered 'yes' to 'Hearing aid user' (Data Field 3393); (4) ICD-10 codes (Data Field 41270): H90, H91.0-H91.9, Z45.3, Z46.1, Z97.4, and Z96.2; (5) ICD-9 code (Data Field 41271): 389. In addition to the exclusion criteria described in Section 2.1.1, participants meeting any of the following conditions were also excluded: (1) failing genotype data quality control, including missingness/heterozygosity > 1.5% (Field ID 22050, 22051); (2) with excessive relatedness among White British samples (Field ID 22012); (3) with discrepancies between genetic sex and self-reported sex (Field ID 22001). The control group was defined as participants who did not meet any inclusion or exclusion criteria and had bilateral SRT of below -5.5 (Field ID 20019, 20021). (Table S2)  Original GWAS datasets of T2D included data from FinnGen[5] and three cohorts from Xue A's study [DIAbetes Genetics Replication and Meta-analysis (DIAGRAM), Genetic Epidemiology Research on Aging (GERA), and UK Biobank][6], encompassing a total of 991,637 European individuals (112,006 cases and 879,631 controls). GWAS of T1D included data from a total of 754,356 European from FinnGen[5] and UK Biobank (10,102 cases and 744,254 controls). |
|  | c) | Describe measurement, quality control and selection of genetic variants | 9 | Additionally, we employed three sensitivity analysis approaches: Cochran’s Q heterogeneity test, MR-Egger pleiotropy test, and MR Pleiotropy Residual Sum and Outlier (MR-PRESSO) pleiotropy test, to verify heterogeneity and pleiotropy of the instrumental variables (IVs). IVs that were considered to be outliers or associated with the outcome (p<0.05) were excluded in the second round of MR to minimize the heterogeneity and pleiotropy. |
|  | d) | For each exposure, outcome, and other relevant variables, describe methods of assessment and diagnostic criteria for diseases | 4,5 (Supplementary Methods) | See Supplementary Methods:  For SNHL, to enhance the statistical power of GWAS and improve the detection of rare variants, we expanded the definition of SNHL in the GWAS analysis. In addition to selecting SNHL participants based on an SRT threshold of -5.5 (Section 2.1.3), other participants meeting at least one of the following criteria were also included as SNHL cases: (1) answered 'yes' to 'Hearing difficulty/problems' (Data Field 2247); (2) answered 'yes' to 'Hearing difficulty/problems with background noise' (Data Field 2257); (3) answered 'yes' to 'Hearing aid user' (Data Field 3393); (4) ICD-10 codes (Data Field 41270): H90, H91.0-H91.9, Z45.3, Z46.1, Z97.4, and Z96.2; (5) ICD-9 code (Data Field 41271): 389. In addition to the exclusion criteria described in Section 2.1.1, participants meeting any of the following conditions were also excluded: (1) failing genotype data quality control, including missingness/heterozygosity > 1.5% (Field ID 22050, 22051); (2) with excessive relatedness among White British samples (Field ID 22012); (3) with discrepancies between genetic sex and self-reported sex (Field ID 22001). The control group was defined as participants who did not meet any inclusion or exclusion criteria and had bilateral SRT of below -5.5 (Field ID 20019, 20021). (Table S2)  Original GWAS datasets of T2D included data from FinnGen[5] and three cohorts from Xue A's study [DIAbetes Genetics Replication and Meta-analysis (DIAGRAM), Genetic Epidemiology Research on Aging (GERA), and UK Biobank][6], encompassing a total of 991,637 European individuals (112,006 cases and 879,631 controls). GWAS of T1D included data from a total of 754,356 European from FinnGen[5] and UK Biobank (10,102 cases and 744,254 controls). |
|  | e) | Provide details of ethics committee approval and participant informed consent, if relevant |  | Not applicable |
| 5 | **Assumptions** | Explicitly state the three core IV assumptions for the main analysis (relevance, independence and exclusion restriction) as well assumptions for any additional or sensitivity analysis | 8,9 | To ensure robustness, the instrumental variables (IVs) in our TSMR analysis must satisfy three assumptions: (1) strong association with the exposure (P < 5 × 10⁻⁸); (2) no association with confounders of the exposure-outcome relationship; (3) association with the outcome exclusively through the exposure. IVs meeting the following criteria were excluded: (1) IVs missing from the GWAS data of the outcomes; (2) palindromic IVs with intermediate allele frequencies (0.42–0.58). For the GSMR study, pleiotropic IVs, identified as heterogeneity in dependent instruments (HEIDI), were excluded. |
| 6 | **Statistical methods: main analysis** | Describe statistical methods and statistics used |  |  |
|  | a) | Describe how quantitative variables were handled in the analyses (i.e., scale, units, model) | 3,5 (Supplementary Methods) | SNHL was treated as a binary variable.  According to the original GWAS studies, T1D and T2D were treated as binary variables. |
|  | b) | Describe how genetic variants were handled in the analyses and, if applicable, how their weights were selected | 8,9 | We conducted a comprehensive bidirectional two-sample MR analysis to identify potential causal relationships, using five methods: inverse variance weighted (IVW), MR-Egger, simple mode, weighted mode, and weighted median. To ensure robustness, the instrumental variables (IVs) in our MR analysis must satisfy three assumptions: (1) strong association with the exposure (P < 5 × 10⁻⁸); (2) no association with confounders of the exposure-outcome relationship; (3) association with the outcome exclusively through the exposure. IVs meeting the following criteria were excluded: (1) IVs missing from the GWAS data of the outcomes; (2) palindromic IVs with intermediate allele frequencies (0.42–0.58). |
|  | c) | Describe the MR estimator (e.g. two-stage least squares, Wald ratio) and related statistics. Detail the included covariates and, in case of two-sample MR, whether the same covariate set was used for adjustment in the two samples | 8 | MR Estimator:  We conducted a comprehensive bidirectional two-sample MR analysis to identify potential causal relationships, using five methods: inverse variance weighted (IVW), MR-Egger, simple mode, weighted mode, and weighted median. |
|  | d) | Explain how missing data were addressed | 9 | IVs meeting the following criteria were excluded: (1) IVs missing from the GWAS data of the outcomes. |
|  | e) | If applicable, indicate how multiple testing was addressed | 9 | For all MR analyses, we maintained a significance threshold of p < 0.05 for individual tests. |
| 7 | **Assessment of assumptions** | Describe any methods or prior knowledge used to assess the assumptions or justify their validity | 9 | Relevance Assumption:  To ensure robustness, the instrumental variables (IVs) in our MR analysis must satisfy three assumptions: (1) strong association with the exposure (P < 5 × 10⁻⁸).  Independence Assumption:  We relied on prior knowledge from the original GWAS studies to ensure that the selected SNPs were not associated with any known confounders (e.g., sociodemographic factors, environmental factors). These studies typically adjust for population structure and key confounders, reducing the risk of confounding bias.  Additionally, we performed Cochran's Q test was used to assess heterogeneity, the MR-Egger intercept test to detect directional pleiotropy, and the MR-PRESSO method to identify and correct for pleiotropic outliers, and any SNPs found to violate the independence assumption were excluded from the analysis.  Exclusion Restriction Assumption:  To assess the exclusion restriction assumption, we used sensitivity analyses to check for horizontal pleiotropy and for potential direct effects of the genetic variants on the outcome through pathways unrelated to the exposure. MR-Egger regression and MR-PRESSO were particularly useful in identifying and addressing potential violations of this assumption.  We also conducted heterogeneity tests (e.g., Cochran's Q test) to assess whether the genetic variants were consistent in their effects on the outcome, which would indicate that they were acting through the exposure rather than other pathways. |
| 8 | **Sensitivity analyses and additional analyses** | Describe any sensitivity analyses or additional analyses performed (e.g. comparison of effect estimates from different approaches, independent replication, bias analytic techniques, validation of instruments, simulations) | 9 | Additionally, we employed three sensitivity analysis approaches: Cochran’s Q heterogeneity test, MR-Egger pleiotropy test, and MR Pleiotropy Residual Sum and Outlier (MR-PRESSO) pleiotropy test, to verify heterogeneity and pleiotropy of the instrumental variables (IVs). IVs that were considered to be outliers or associated with the outcome (p<0.05) were excluded in the second round of MR to minimize the heterogeneity and pleiotropy. |
| 9 | **Software and pre-registration** |  |  |  |
|  | a) | Name statistical software and package(s), including version and settings used | 9 | The statistical analysis process for MR was conducted using the TwoSampleMR package in R version 3.6.3. For all MR analyses, we maintained a significance threshold of p < 0.05 for individual tests. |
|  | b) | State whether the study protocol and details were pre-registered (as well as when and where) |  | Not applicable |
|  | **RESULTS** |  |  |  |
| 10 | **Descriptive data** |  |  |  |
|  | a) | Report the numbers of individuals at each stage of included studies and reasons for exclusion. Consider use of a flow diagram | 4,5 (Supplementary Methods) | Finally, we identified 112,363 SNHL cases and 60,744 controls.  Original GWAS datasets of T2D included data from FinnGen[5] and three cohorts from Xue A's study [DIAbetes Genetics Replication and Meta-analysis (DIAGRAM), Genetic Epidemiology Research on Aging (GERA), and UK Biobank][6], encompassing a total of 991,637 European individuals (112,006 cases and 879,631 controls). GWAS of T1D included data from a total of 754,356 European from FinnGen[5] and UK Biobank (10,102 cases and 744,254 controls). According to the original GWAS studies, T1D and T2D were treated as binary variables. Study-specific characteristics, quality control measures, and descriptive statistics for these datasets can be found in previously published research[7, 8]. Sample-size-weighted meta-analyses were performed for both T2D and T1D to increase statistical power of genetic variants. |
|  | b) | Report summary statistics for phenotypic exposure(s), outcome(s), and other relevant variables (e.g. means, SDs, proportions) | 19 | After excluding outcome-related IVs and outliers (T2D: from 187 IVs to 169 IVs; SNHL: from 7 IVs to 6 IVs) (Table S14 and Table S15), the IVs in the second round of forward and reverse MR analysis exhibited minimal heterogeneity and pleiotropy (Table S16). |
|  | c) | If the data sources include meta-analyses of previous studies, provide the assessments of heterogeneity across these studies | 5 (Supplementary Methods) | Study-specific characteristics, quality control measures, and descriptive statistics for these datasets can be found in previously published research[7, 8]. Sample-size-weighted meta-analyses were performed for both T2D and T1D to increase statistical power of genetic variants. |
|  | d) | For two-sample MR:  i.  Provide justification of the similarity of the genetic variant-exposure associations between the exposure and outcome samples  ii.  Provide information on the number of individuals who overlap between the exposure and outcome studies | 9 | Considering the overlap of UK Biobank samples between T2D GWAS study (Xue A et al.) and the SNHL GWAS study, we used only the T2D GWAS summary statistics from FinnGen for a bidirectional causal effect MR analysis between T2D and SNHL, in order to minimize false positive results[9]. |
| 11 | **Main results** |  |  |  |
|  | a) | Report the associations between genetic variant and exposure, and between genetic variant and outcome, preferably on an interpretable scale | 8 | To ensure robustness, the instrumental variables (IVs) in our TSMR analysis must satisfy three assumptions: (1) strong association with the exposure (P < 5 × 10⁻⁸). |
|  | b) | Report MR estimates of the relationship between exposure and outcome, and the measures of uncertainty from the MR analysis, on an interpretable scale, such as odds ratio or relative risk per SD difference | 19 | However, among the five methods applied, only the IVW approach suggested a potential positive causal relationship from T2D to SNHL (b = 0.02, p = 0.05), whereas the other methods did not identify any forward or reverse causal relationship between T2D and SNHL. (Table S16) |
|  | c) | If relevant, consider translating estimates of relative risk into absolute risk for a meaningful time period |  | Not applicable |
|  | d) | Consider plots to visualize results (e.g. forest plot, scatterplot of associations between genetic variants and outcome versus between genetic variants and exposure) | Table S16 | Not applicable |
| 12 | **Assessment of assumptions** |  |  |  |
|  | a) | Report the assessment of the validity of the assumptions | 15 | The mean F-statistic for the IVs in TSMR exceeded 100, which suggested that the genetic variants used as instruments had a robust association with the exposure. Sensitivity analyses revealed no heterogeneity among IVs based on the Cochran Q heterogeneity test. Although the Egger intercept suggested potential pleiotropy, MR-PRESSO did not identify any pleiotropic outliers, indicating minimal pleiotropy and robust results. (Table S5). |
|  | b) | Report any additional statistics (e.g., assessments of heterogeneity across genetic variants, such as *I^2^*, Q statistic or E-value) | 19 | After excluding outcome-related IVs and outliers (T2D: from 187 IVs to 169 IVs; SNHL: from 7 IVs to 6 IVs) (Table S14 and Table S15), the IVs in the second round of forward and reverse MR analysis exhibited minimal heterogeneity and pleiotropy (Table S16). |
| 13 | **Sensitivity analyses and additional analyses** |  |  |  |
|  | a) | Report any sensitivity analyses to assess the robustness of the main results to violations of the assumptions | 19 | After excluding outcome-related IVs and outliers (T2D: from 187 IVs to 169 IVs; SNHL: from 7 IVs to 6 IVs) (Table S14 and Table S15), the IVs in the second round of forward and reverse MR analysis exhibited minimal heterogeneity and pleiotropy (Table S16). |
|  | b) | Report results from other sensitivity analyses or additional analyses | 19 | After excluding outcome-related IVs and outliers (T2D: from 187 IVs to 169 IVs; SNHL: from 7 IVs to 6 IVs) (Table S14 and Table S15), the IVs in the second round of forward and reverse MR analysis exhibited minimal heterogeneity and pleiotropy (Table S16). |
|  | c) | Report any assessment of direction of causal relationship (e.g., bidirectional MR) | 19 | However, among the five methods applied, only the IVW approach suggested a potential positive causal relationship from T2D to SNHL (b = 0.02, p = 0.05), whereas the other methods did not identify any forward or reverse causal relationship between T2D and SNHL. (Table S16) |
|  | d) | When relevant, report and compare with estimates from non-MR analyses | 22 | Unlike some phenotypic association analysis[1-4], MR analysis indicated no compelling causal relationship between T2D and SNHL, supporting the hypothesis that their comorbidity was partially driven by a shared genetic basis. |
|  | e) | Consider additional plots to visualize results (e.g., leave-one-out analyses) |  | Not applicable |
|  | **DISCUSSION** |  |  |  |
| 14 | **Key results** | Summarize key results with reference to study objectives | 22 | Unlike some phenotypic association analysis[1-4], MR analysis indicated no compelling causal relationship between T2D and SNHL, supporting the hypothesis that their comorbidity was partially driven by a shared genetic basis. |
| 15 | **Limitations** | Discuss limitations of the study, taking into account the validity of the IV assumptions, other sources of potential bias, and imprecision. Discuss both direction and magnitude of any potential bias and any efforts to address them | 25 | Third, though MR results indicate that the observed association and genetic overlap is more likely due to pleiotropy rather than causality, further studies with larger sample sizes are needed to confirm these findings. |
| 16 | **Interpretation** |  |  |  |
|  | a) | Meaning: Give a cautious overall interpretation of results in the context of their limitations and in comparison with other studies | 22,25 | Unlike some phenotypic association analysis[1-4], MR analysis indicated no compelling causal relationship between T2D and SNHL, supporting the hypothesis that their comorbidity was partially driven by a shared genetic basis.  Third, though MR results indicate that the observed association and genetic overlap is more likely due to pleiotropy rather than causality, further studies with larger sample sizes are needed to confirm these findings. |
|  | b) | Mechanism: Discuss underlying biological mechanisms that could drive a potential causal relationship between the investigated exposure and the outcome, and whether the gene-environment equivalence assumption is reasonable. Use causal language carefully, clarifying that IV estimates may provide causal effects only under certain assumptions |  | Not applicable because no compelling causal relationship was shown. |
|  | c) | Clinical relevance: Discuss whether the results have clinical or public policy relevance, and to what extent they inform effect sizes of possible interventions | 22 | Unlike some phenotypic association analysis[1-4], MR analysis indicated no compelling causal relationship between T2D and SNHL, supporting the hypothesis that their comorbidity was partially driven by a shared genetic basis. |
| 17 | **Generalizability** | Discuss the generalizability of the study results (a) to other populations, (b) across other exposure periods/timings, and (c) across other levels of exposure | 24 | This study has several limitations. First, the data were sourced from European populations, which might limit the generalizability of the findings, and future studies should include more diverse populations. |
|  | **OTHER INFORMATION** |  |  |  |
| 18 | **Funding** | Describe sources of funding and the role of funders in the present study and, if applicable, sources of funding for the databases and original study or studies on which the present study is based | 30 | Funding  This work was supported by West China Hospital, Sichuan University (ZY, grant #ZYJC21027); Sichuan University (PWD, grant: 2023HXBH119); Chengdu Science and Technology Bureau (ZY, grant #2022-YF05-01387-SN); The Science and Technology Department of Sichuan Province (ZY, grant #2024YFHZ0076; PWD, grant #2025ZNSFSC1526); The Foundation of National Clinical Research Center for Geriatrics (ZY, grant #Z2023LC002); National Natural Youth Science Foundation of China (PWD, grant #82301307); Chengdu Science and Technology Bureau (ZY, grant# 2024-YF05-00050-SN); Health Care Research Project of Sichuan Province (ZY, grant#ZH2025-102); National Key Research and Development Program of China (ZY, grant #2024YFC2418305). |
| 19 | **Data and data sharing** | Provide the data used to perform all analyses or report where and how the data can be accessed, and reference these sources in the article. Provide the statistical code needed to reproduce the results in the article, or report whether the code is publicly accessible and if so, where | 29,30 | Availability of data and materials  The relevant data for the observational study from the UK Biobank cohort can be accessed with permission from the UK Biobank (https://www.ukbiobank.ac.uk/enable-your-research/apply-for-access). Genetic data for T1D and T2D were obtained from FinnGen (https://www.finngen.fi/en/access_results), the study by Xue et al. (DOI: 10.1038/s41467-018-04951-w; available via the IEU OpenGWAS project: https://gwas.mrcieu.ac.uk/datasets/ebi-a-GCST006867/), and the study by Joshua Chiou et al. (DOI: 10.1038/s41586-021-03552-w; accessible through the GWAS Catalog: https://www.ebi.ac.uk/gwas/studies/GCST90014023). |
| 20 | **Conflicts of Interest** | All authors should declare all potential conflicts of interest | 29 | Competing interests  The authors declare that they have no competing interests. |

This checklist is copyrighted by the Equator Network under the Creative Commons Attribution 3.0 Unported (CC BY 3.0) license.

Reference

1.	Gioacchini, F.M., et al., *Diabetes Mellitus and Hearing Loss: A Complex Relationship.* Medicina (Kaunas), 2023. **59**(2).

2.	Gupta, S., et al., *Type 2 diabetes and the risk of incident hearing loss.* Diabetologia, 2019. **62**(2): p. 281-285.

3.	Hong, O., J. Buss, and E. Thomas, *Type 2 diabetes and hearing loss.* Dis Mon, 2013. **59**(4): p. 139-46.

4.	Williams, E.D. and M.E. Rubio, *Associations between diabetes mellitus and sensorineural hearing loss from humans and animal studies.* Hear Res, 2024. **450**: p. 109072.

5.	Kurki, M.I., et al., *FinnGen provides genetic insights from a well-phenotyped isolated population.* Nature, 2023. **613**(7944): p. 508-518.

6.	Xue, A., et al., *Genome-wide association analyses identify 143 risk variants and putative regulatory mechanisms for type 2 diabetes.* Nat Commun, 2018. **9**(1): p. 2941.

7.	Willer, C.J., Y. Li, and G.R. Abecasis, *METAL: fast and efficient meta-analysis of genomewide association scans.* Bioinformatics, 2010. **26**(17): p. 2190-1.

8.	Xiao, L., et al., *The interactions between host genome and gut microbiome increase the risk of psychiatric disorders: Mendelian randomization and biological annotation.* Brain Behav Immun, 2023. **113**: p. 389-400.

9.	Yuan, S., et al., *Smoking, alcohol consumption, and 24 gastrointestinal diseases: Mendelian randomization analysis.* Elife, 2023. **12**.
