# Supplementary material for: Conventional and Shared Genetic Association Analysis Between Diabetes Mellitus and Sensorineural Hearing Loss
Source: Hum Mutat. 2026 Sep 15;2026:5099141. doi: 10.1155/humu/5099141 (PMC13576905; doi:10.1155/humu/5099141)
Supplement: Supplementary file 2 [file HUMU-2026-5099141-s003.docx]

1. **Phenotypic association analyses between DM and SNHL**
   1.1. **Definition of DM and SNHL**

The participants meeting either of following conditions were considered as total diabetes: 1) Individuals who reported themselves taking Insulin in a self-reported questionnaire (Field ID=6177, 6153); 2) Participants with hospital inpatient admissions according to ICD-9 codes: E10-E14; 3) Patients diagnosed with diabetes by a doctor; 4) Individuals who self-report having diabetes (Field ID=20002).

As for T1D, participants under these circumstances would be considered as patients of this type: 1) People that claimed themselves for suffering from T1D. (Field ID=20002); 2) Participants having ICD-10 records of T1D: E10; 3) Individuals who were diagnosed with diabetes by a doctor (Field ID=2443) when aged less than 30 (Field ID=2976), while using insulin within one year diagnosis of diabetes (Field ID=2986)^1^.

For patients with T2D, they all met the criteria below: 1) Participants with records of T2D according to ICD-10 codes: E11; 2) Individuals who reported to have T2D in a self-reported questionnaire; 3) Patients that were diagnosed T2D by a doctor (Field ID=2443) after the age of 35 (Field ID=2976) and didn’t start taking insulin within one year after diagnosis of diabetes (Field ID=2986)^2^.

DM patients could further be classified into the following four levels of hemoglobin A1c (HbA1c) according to concentration in peripheral blood: low-HbA1c (<31 mmol/mol), normal-HbA1c (between 31~<39 mmol/mol), increased-HbA1c (between 39~<48) and high- HbA1c (≥48mmol/mol)^3^.

SNHL was identified by speech-reception-threshold (SRT) estimate. It should be noted that we mainly focus on SNHL, and that conductive and mixed ones have been excluded from the study. Both sides of ears were tested and recorded separately (Field ID=20019 for outcomes of left ear and 20021 for outcomes of right ear). Two definitions were used to identify total SNHL: 1) When SRT data was available for both sides of ears, either side with an SRT≥-5.5 dB was considered as hearing loss. 2) When data was available for only one side, it was considered as hearing loss regardless of whether the other side was good or not. This is because that the absence of SRT data is not due to missing data, but rather because the patient reported being unable to hear any sound in that ear before the test, rendering them unable to complete the test^4^.

SNHL was categorized into two subtypes: unilateral SNHL and bilateral SNHL. When a patient had data for both ears, and that the SRT for both sides were≥-5.5 dB, or when there was data for only one ear, and the SRT for that ear was≥-5.5 dB, they were considered as bilateral SNHL patients. Participants with unilateral SNHL either had data for both ears, with the SRT of one ear≥-5.5dB and the other ear < -5.5dB, or they had data for only one ear, with the SRT of that ear <-5.5dB. Conversely, “normal” participants referred to the individuals without SNHL, who had data for both ears, and the SRT were both < -5.5dB. Due to the fact that unilateral hearing loss has a relatively smaller impact on overall auditory function, we classified the severity of hearing loss only for bilateral cases. In the case of bilateral SNHL, using the better ear as a reference, we divided it into three degrees at -5.5 dB and -3.5 dB thresholds. The "Normal" degree included those individuals whose SRT was < -5.5 dB, the "Insufficient" degree represents those participants whose SRT was between -5.5 dB and -3.5 dB, and the "Poor" degree are for patients whose SRT was > -3.5 dB^5^.

The association between diabetes mellitus and bilateral SNHL was then analyzed in stratified participants based on various levels (normal/insufficient/poor) of HL in all types of DM participants, including total diabetes, T1D and T2D.

1.2. **Information of covariate**

The covariates included age, sex, education background, current employment status (retired/unemployed/working/others), body mass index (BMI, under-weight/normal weight/over-weight/obese), ethnic background (white/others), smoking status (never/only previous/ currently), alcohol drinking status (never/only previous/currently), occupation noise exposure, and cardiovascular diseases, which consisted of coronary atherosclerosis, angina pectoris, myocardial infarction, stroke, subarachnoid hemorrhage and cerebral hemorrhage.

2. **Shared genetic architecture between DM and SNHL**
   2.1. **GWAS resources and meta-analysis**

SNHL was treated as a binary variable. To enhance the statistical power of GWAS and improve the detection of rare variants, we expanded the definition of SNHL in the GWAS analysis. In addition to selecting SNHL participants based on an SRT threshold of -5.5 **(Section 2.1.3)**, other participants meeting at least one of the following criteria were also included as SNHL cases: (1) answered “yes” to “Hearing difficulty/problems” (Data Field 2247); (2) answered “yes” to “Hearing difficulty/problems with background noise” (Data Field 2257); (3) answered “yes” to “Hearing aid user” (Data Field 3393); (4) ICD-10 codes (Data Field 41270): H90, H91.0-H91.9, Z45.3, Z46.1, Z97.4, and Z96.2; (5) ICD-9 code (Data Field 41271): 389. In addition to the exclusion criteria described in **Section 2.1.1**, participants meeting any of the following conditions were also excluded: (1) failing genotype data quality control, including missingness/heterozygosity > 1.5% (Field ID 22050, 22051); (2) with excessive relatedness among White British samples (Field ID 22012); (3) with discrepancies between genetic sex and self-reported sex (Field ID 22001). The control group was defined as participants who did not meet any inclusion or exclusion criteria and had bilateral SRT of below -5.5 (Field ID 20019, 20021) **(Table S2).** Finally, we identified 112,363 SNHL cases and 60,744 controls.

We obtained the genotype data for the aforementioned participants from the UK BiLEVE Axiom array and the Affymetrix UK Biobank Axiom array. These genotype data were quality-controlled, with the following exclusions applied: (1) SNPs with a high genotype missingness rate (>10%); (2) SNPs with a minor allele frequency (MAF) < 0.01; and (3) SNPs significantly deviating from Hardy-Weinberg equilibrium (HWE) (p < 1 × 10⁻¹⁵). Association analyses were performed using REGENIE (Version 2.0.2), a robust and efficient method for genome-wide association studies. Covariates including age, sex, body mass index (BMI), and 20 ancestry principal components were adjusted to account for potential confounding effects.

Original GWAS datasets of T2D included data from FinnGen^6^ and three cohorts from Xue A's study [DIAbetes Genetics Replication and Meta-analysis (DIAGRAM), Genetic Epidemiology Research on Aging (GERA), and UK Biobank]^7^, encompassing a total of 991,637 European individuals (112,006 cases and 879,631 controls). GWAS of T1D included data from a total of 754,356 European from FinnGen^6^ and UK Biobank (10,102 cases and 744,254 controls). According to the original GWAS studies, T1D and T2D were treated as binary variables. Study-specific characteristics, quality control measures, and descriptive statistics for these datasets can be found in previously published research^8,9^. Sample-size-weighted meta-analyses were performed for both T2D and T1D to increase statistical power of genetic variants.

2.2. **Global and local genetic correlation analyses of DM and SNHL**

We applied LDSC and Stratified LDSC for the calculations global and local genetic correlations. LDSC is based on the relationship between LD score and Chi-square statistics, which are fitted by linear regression.

For local genetic correlations, a total of 28 functional categories were enrolled, and were further classified and arranged in eight regions:

Chromatin Marks: this category includes histone modification marks, such as H3K27ac Hnisz, H3K9ac Trynka, H3K4me1 Trynka, H3K4me3 Trynka, H3K27ac PGC2, H3K9ac peaks Trynka, H3K4me1 peaks Trynka, and H3K4me3 peaks Trynka.

Promoters and Enhancers: this group comprises regulatory elements associated with gene activation, including Promoter UCSC, SuperEnhancer Hnisz, Enhancer Hoffman, WeakEnhancer Hoffman, PromoterFlanking Hoffman, and Enhancer Andersson.

DNA Binding Sites: this category consists of transcription factor binding sites and chromatin interaction regions, including TFBS ENCODE and CTCF Hoffman.

Open Chromatin Regions: these regions indicate accessible chromatin states and include DHS Trynka, DHS peaks Trynka, DGF ENCODE, and FetalDHS Trynka.

Coding Regions: these regions are directly involved in gene expression and include Coding UCSC, UTR 5 UCSC, UTR 3 UCSC, and Intron UCSC.

Transcriptional Regions: these regions are associated with transcriptional activity and include TSS Hoffman and Transcribed Hoffman.

Repressed Regions: this category includes regions associated with transcriptional repression, represented by Repressed Hoffman.

Conserved Regions: this category represents evolutionarily conserved sequences, including Conserved LindbladToh.

2.3. **Genetic causality inference analysis of T2D and SNHL**

To ensure robustness, the instrumental variables (IVs) in our MR analysis must satisfy three assumptions: (1) strong association with the exposure (P < 5 × 10⁻⁸); (2) no association with confounders of the exposure-outcome relationship; (3) association with the outcome exclusively through the exposure. IVs meeting the following criteria were excluded: (1) IVs missing from the GWAS data of the outcomes; (2) palindromic IVs with intermediate allele frequencies (0.42–0.58).

Additionally, we employed three sensitivity analysis approaches: Cochran’s Q heterogeneity test, MR-Egger pleiotropy test, and MR Pleiotropy Residual Sum and Outlier (MR-PRESSO) pleiotropy test, to verify heterogeneity and pleiotropy of the instrumental variables (IVs). IVs that were considered to be outliers or associated with the outcome (p<0.05) were excluded in the second round of MR to minimize the heterogeneity and pleiotropy.

Considering the overlap of UK Biobank samples between T2D GWAS study (Xue A et al.) and the SNHL GWAS study, we used only the T2D GWAS summary statistics from FinnGen for a bidirectional causal effect MR analysis between T2D and SNHL, in order to minimize false positive results^10^.

The statistical analysis process for MR was conducted using the TwoSampleMR package in R version 3.6.3. For all MR analyses, we maintained a significance threshold of p < 0.05 for individual tests.

2.4. **Identification of pleiotropic loci for SNHL-T2D by cross-trait meta-analysis**

Based on the effect sizes (β) and standard errors (SE) of SNPs from GWAS of SNHL and T2D, MTAG was performed using the generalized method of moments to calculated variance weights and identify shared genetic loci between traits. Additionally, CPASSOC, another independent method for exploring the pleiotropic genetic basis, were conducted to doubly validate the shared loci^11^. P-value thresholds of both P_MTAG_ and P_CPASSOC_<5×10−8 were considered to satisfy the criteria for the pleiotropic SNP and locus.

Functional mapping and annotation of GWAS (FUMA, https://fuma.ctglab.nl, version 1.5.4) was used to perform clumping on the cross-trait GWAS summary data. The analysis was based on the 1000 Genomes Project (Phase 3 EUR) data, with an r² threshold of 0.6 employed to distinguish independent loci, and an r² threshold of 0.1 used to identify lead SNPs.

Based on reference data from the 1000 Genomes Project (Phase 3 EUR), fine-mapping utilized the LD structure between SNPs to calculate the posterior probability of candidate SNPs being causal variants. This approach was used to validate whether the lead SNPs inferred by MTAG were credible shared variants.

2.5. **Gene annotation and priority for SNHL-T2D**

Five methods were applied to jointly infer the protein-coding genes associated with the lead SNPs of HL-T2D identified by MTAG and CPASSOC: (1) nearest location, (2) expression quantitative trait loci (eQTL) colocalization, (3) high-throughput chromatin conformation capture (Hi-C) colocalization, (4) MAGMA genes, and (5) Summary-based Mendelian Randomization (SMR) genes.

The first four analyses were further conducted using FUMA. (1) The nearest gene referred to the gene which located within or closest to the loci. (2) eQTL genes involved statistical associations between SNPs and gene expression levels from Genotype-Tissue Expression project (GTEx) tissues related to hearing (arteries and blood, simulating vascularized tissue of the inner ear), as well as metabolism-related tissues (fat, liver, pancreas, muscle). (3) Hi-C captured the three-dimensional interactions of DNA in the chromosomal space, identifying spatial interactions between the region where the lead SNP was located and the promoter regions of genes, helping to infer SNP regulation of distal genes. (4) If a SNP is located within a gene, MAGMA (version 1.0.8) used multiple linear regression to calculate gene-based p-values for protein-coding genes by mapping the SNP to the gene^12^. Only genes with both a P_MAGMA_ value less than 0.05/18,622 in MTAG (based on 18,622 genes) and less than 0.05/19,084 in CPASSOC (based on 19,084 genes) were considered significantly associated with the shared traits. (5) SMR was a MR method based on summary-level statistical data, integrating eQTL information in blood from eQTLgen (https://www.eqtlgen.org/) with GWAS data from MTAG to explore causal relationships between genes and the shared phenotypes of T2D-SNHL. To determine whether the association between gene expression and phenotypes was influenced by pleiotropy from the same SNP, SMR employed the heterogeneity in dependent instrument (HEIDI) test. Genes with P_smr_ less than 0.05 and P_HEIDI_ greater than 0.01 were considered causal genes for the shared phenotype of HL-T2D^13^.

**Reference**

1.	Sticht J, Álvaro-Benito M, Konigorski S. Type 1 Diabetes and the HLA Region: Genetic Association Besides Classical HLA Class II Genes. *Front Genet*. 2021;12:683946. doi:10.3389/fgene.2021.683946

2.	Tyrrell JS, Yaghootkar H, Freathy RM, Hattersley AT, Frayling TM. Parental diabetes and birthweight in 236 030 individuals in the UK biobank study. *Int J Epidemiol*. Dec 2013;42(6):1714-23. doi:10.1093/ije/dyt220

3.	Peila R, Rohan TE. Diabetes, Glycated Hemoglobin, and Risk of Cancer in the UK Biobank Study. *Cancer Epidemiol Biomarkers Prev*. Jun 2020;29(6):1107-1119. doi:10.1158/1055-9965.Epi-19-1623

4.	Machado-Fragua MD, Struijk EA, Yévenes-Briones H, Caballero FF, Rodríguez-Artalejo F, Lopez-Garcia E. Coffee consumption and risk of hearing impairment in men and women. *Clin Nutr*. May 2021;40(5):3429-3435. doi:10.1016/j.clnu.2020.11.022

5.	Dawes P, Fortnum H, Moore DR, et al. Hearing in middle age: a population snapshot of 40- to 69-year olds in the United Kingdom. *Ear Hear*. May-Jun 2014;35(3):e44-51. doi:10.1097/aud.0000000000000010

6.	Kurki MI, Karjalainen J, Palta P, et al. FinnGen provides genetic insights from a well-phenotyped isolated population. *Nature*. Jan 2023;613(7944):508-518. doi:10.1038/s41586-022-05473-8

7.	Xue A, Wu Y, Zhu Z, et al. Genome-wide association analyses identify 143 risk variants and putative regulatory mechanisms for type 2 diabetes. *Nat Commun*. Jul 27 2018;9(1):2941. doi:10.1038/s41467-018-04951-w

8.	Willer CJ, Li Y, Abecasis GR. METAL: fast and efficient meta-analysis of genomewide association scans. *Bioinformatics*. Sep 1 2010;26(17):2190-1. doi:10.1093/bioinformatics/btq340

9.	Xiao L, Liu S, Wu Y, et al. The interactions between host genome and gut microbiome increase the risk of psychiatric disorders: Mendelian randomization and biological annotation. *Brain Behav Immun*. Oct 2023;113:389-400. doi:10.1016/j.bbi.2023.08.003

10.	Yuan S, Chen J, Ruan X, et al. Smoking, alcohol consumption, and 24 gastrointestinal diseases: Mendelian randomization analysis. *Elife*. Feb 2 2023;12doi:10.7554/eLife.84051

11.	Zhu X, Feng T, Tayo BO, et al. Meta-analysis of correlated traits via summary statistics from GWASs with an application in hypertension. *Am J Hum Genet*. Jan 8 2015;96(1):21-36. doi:10.1016/j.ajhg.2014.11.011

12.	Watanabe K, Taskesen E, van Bochoven A, Posthuma D. Functional mapping and annotation of genetic associations with FUMA. *Nat Commun*. Nov 28 2017;8(1):1826. doi:10.1038/s41467-017-01261-5

13.	Fu K, Si S, Jin X, et al. Exploring antidiabetic drug targets as potential disease-modifying agents in osteoarthritis. *EBioMedicine*. Sep 2024;107:105285. doi:10.1016/j.ebiom.2024.105285
