# Supplementary material for: Conventional and Shared Genetic Association Analysis Between Diabetes Mellitus and Sensorineural Hearing Loss
Source: Hum Mutat. 2026 Sep 15;2026:5099141. doi: 10.1155/humu/5099141 (PMC13576905; doi:10.1155/humu/5099141)
Supplement: Supplementary file 3 [file HUMU-2026-5099141-s004.docx]

***Figure S1. Phenotypic association between DM and SNHL across age and sex subgroups***

***Figure S2. Manhattan plot of summary results from cross-trait meta-analyses of T2D-SNHL.***

***Figure S3. QQ plot of summary results from cross-trait meta-analyses of T2D-SNHL.***

***Figure S4. MAGMA genes of summary results from cross-trait meta-analyses of T2D-SNHL.***

***Figure S5. Enrichment results of cross-trait meta-analyses of T2D-SNHL in 54 GTEx tissues.***

***Figure S6. Enrichment of shared genes for T2D-SNHL across 54 GTEx tissues***
